# Genetic characterization of Porcine Circovirus 2 found in Malaysia

**DOI:** 10.1186/1743-422X-8-437

**Published:** 2011-09-13

**Authors:** Seetha Jaganathan, Ooi P Toung, Phang L Yee, Tan D Yew, Choo P Yoon, Lim B Keong

**Affiliations:** 1 Department of Clinical Studies, Faculty of Veterinary Medicine, Universiti Putra Malaysia, 43400 UPM, Serdang, Selangor, Malaysia; 2 Department of Biotechnology, Faculty of Biotechnology, Universiti Putra Malaysia, 43400 UPM, Serdang, Selangor, Malaysia; 3 Asia-Pacific Special Nutrients Sdn. Bhd, Lot 18B, Jalan 241, Section 51A, 46100 Petaling Jaya, Selangor, Malaysia; 4 Rhone Ma Malaysia (M) Sdn Bhd, Lot 18B, Jalan 241, Section 51A, 46100, Petaling Jaya, Selangor, Malaysia

**Keywords:** PCV2, PCVAD, phylogenetic

## Abstract

**Background:**

Porcine circovirus type 2 is the primary etiological agent associated with a group of complex multi-factorial diseases classified as Porcine Circovirus Associated Diseases (PCVAD). Sporadic cases reported in Malaysia in 2007 caused major economic losses to the 2.2 billion Malaysian ringgit (MYR) (approximately 0.7 billion US dollar) swine industry. The objective of the present study was to determine the association between the presence of PCV2 and occurrences of PCVAD.

**Results:**

This study showed that 37 out of 42 farms sampled were positive for PCV2 using PCR screening. Thirteen whole genome of PCV2 isolates from pigs with typical PCVAD symptoms were successfully sequenced. These isolates shared 98.3-99.2% similarities with sequences of isolates from the Netherlands. All thirteen isolates fell into the same clade as PCV2b isolates from other countries. Amino acid sequence analysis of the putative capsid protein (ORF2) of the PCV2 revealed that there are three clusters found in Malaysia, namely cluster 1C and 1A/1B. Of interest, three of the isolates (isolates Mal 005, Mal 006 and Mal 010) had a proline substitution for arginine or isoleucine encoded at nt. position 88-89. Eight of the isolates had mutations at the C terminus of the putative capsid protein suggestive of higher pathogenicity which may account for the high reports of PCVAD clinical symptoms in 2007.

**Conclusion:**

Phylogenetic study suggests that there may be a link between movements of animals by import of breeders into the country being the route of entry of the virus. While it is not possible to eradicate the virus from commercial pigs, the swine industry in Malaysia can be safeguarded by control measures implemented throughout the country. These measures should include improved biosecurity, disease surveillance; vaccination as well as enforcement of regulations formulated to control and prevent the spread of this disease on a national scale.

## Background

Porcine circovirus (PCV) is a small non-enveloped DNA virus with a single-stranded circular DNA and a genome of about 1.76 kbp, making it the smallest autonomously replicating virus [1-6]. Porcine circovirus was first isolated as a non-cytopathic contaminant of the porcine kidney cell line PK15 in 1974 [7-9] and since then has been associated with many diseases related to swine. There are several genotypes of PCV. The most commonly reported genotypes are PCV1 and PCV2 [10-12]. However, a new PCV genotype was recently documented in Canada which contains the ORF1 of PCV1 and the ORF2 of PCV2a. This new genotype which was assigned PCV1/2a has so far only been reported in Canada [13]. Within the PCV2 genotype, there are several sub-types (PCV2a-2e). PCV2c has been reported in pigs from Denmark [14], PCV2d are dominant in pigs from China [15] and PCV2e are found in pigs from Thailand [16]. Although PCV1 and PCV2 show high levels of nucleotide similarity, PCV1 is considered non-pathogenic whilst PCV2 has been reported as pathogenic and has been identified as the causative agent of PCV-associated diseases (PCVAD) or sometimes also referred to as postweaning multisystemic wasting syndrome (PMWS), a term used to describe all PCV2-associated clinical and subclinical manifestations [17-23]. Genetic characterizations based on the sequences that encode the putative capsid protein, (ORF2) has also identified 2 groups of PCV2 genome with a total of eight clusters (1A to 1C and 2A to 2E) [11,24,25]. Group 1 has the sequence CCCCG/TC which encodes for proline and arginine/leucine (PR/PL) at nt. position 88-89, while group 2 has the sequence AAAATC which encodes for lysine and isoleucine (KI) at nt. position 88-89 [26]. It is generally understood and accepted that group 1 represents PCV2b whilst group 2 represents PCV2a. Olvera et al. [26] reported genomes belonging to cluster 1B could be the product of a recombinant event between a genome of cluster 1A as the major parent and a genome of group 2 (most probably belonging to cluster 2D) as the minor parent [26] with PCV2b being the most prevalent form displaying highest pathogenicity compared to other genotypes [27]. Mutations have also been reported in many PCV2 strains worldwide including elongations in the putative capsid protein as a result of a single lysine (K) residue deletions at the C-terminus of the putative capsid protein in the stop codon of the open reading frame 2 (ORF2) [15,24,26,28]. This could account for the variations in the PCV2 findings reported worldwide in particular, the recent PCV2 studies where several new genotypes have been reported in Canada [13], Denmark [14], China [15] and Thailand [16]. Overall, both genotypes share less than 80% nucleotide sequence homology and approximately 75% homology at the amino-acid level [5,22,25,29-32].

In Malaysia, PCV2 was first identified by the Veterinary Research Institute in 2004 using RFLP methods followed by the first case study of PCVAD in 2007 based on clinical features, histopathology findings and PCR screening [33]. Globally, the virus has been reported in pigs in Canada and the United States, several European countries and some countries in Asia and can be considered as one of the most economically important emerging swine pathogens [14,15,19,26,34-38]. Realizing the impact it has on the swine industry in Malaysia which is worth over 2.2 billion ringgit, there is a need to understand the genetic characteristics of PCV2 to monitor its distribution. By understanding the genetic presence of PCV2 and its relationship to PCVAD, veterinary practitioners may be able to develop better vaccination programs against the disease. Currently, there are very few publications about PCV2 in this country. This study represents the complete genetic characterization and phylogenetic analysis of PCV2 reported for the first time in Malaysia.

42 pig farms were screened for PCV1 and PCV2. Thirteen positive samples from pigs with confirmed PCV2 were genetically characterized. These samples were from different states in Malaysia. Genetic variation among isolates from this study and PCV2 sequences from other countries deposited in Genbank were analyzed and compared.

## Results

### The detection of PCV1 and PCV2 from the Malaysian pig herd

Samples were collected from animals with typical clinical signs of PCVAD which include enlarged lymph nodes, wasting, dyspnea, pallor, severe weight loss and jaundice [37]. Samples tested positive for PCV1 were not used for further analysis. Initial investigations revealed that 37 out of the 42 farms were positive for PCV2 by conventional PCR.

### Genetic characterization, amino acid sequence analysis and phylogenetic study of the PCV2 isolates in Malaysia

**Figure 1:**
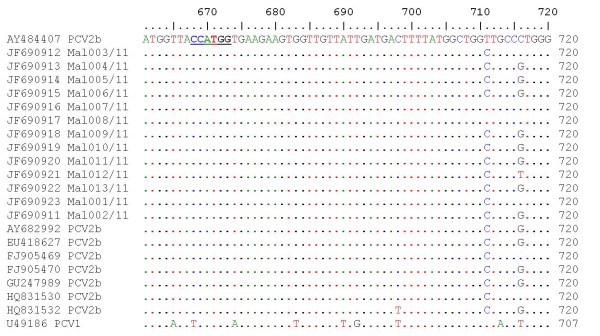
**Nucleotide sequence alignment of a fragment of the complete genome of PCV1 and PCV2 found in this study**. Alignments were performed using ClustalW. The unique *NcoI *restriction enzyme site that is present in all PCV2 [10] isolates is underlined.

A preliminary BLAST analysis confirmed that all 13 isolates were PCV2. As reported previously, the PCV genomic sequences ranged from 1758 to 1768 bp [25,38]. Assembly of the complete genomes of all 13 PCV2 isolates showed that all isolates from this study were 1767 bp in length. To confirm that all isolates from this study have the unique *Nco*I restriction enzyme site that exist only in PCV2 [10,38] bioinformatics analysis was employed to map the restriction enzyme sites present in the complete genome. Our analysis confirmed that this unique feature was present in all the complete genomes isolated in this study at nucleotide position 668 to 673 (Figure 1). Phylogenetic analysis showed that all 13 isolates used for genetic characterization formed a group with other PCV2b isolates. Amino acid sequence analysis showed 3 distinct clusters; cluster 1C and cluster 1A/1B.

## Discussion

Based on the 2010 data provided by the Department of Veterinary Services (DVS) on their website, there are approximately 565 pig farms in Malaysia. There are a total of 14 states in Malaysia. The 3 states of Kelantan, Terengganu and Pahang do not have any pig farms whilst the states of Pahang, Perlis and Negeri Sembilan have only 1 pig farm each and Kedah has only 2 pig farms. Therefore, samples in this study were collected from 6 states with big farms that are key players in the swine industry to represent the big picture of PCV2 distribution in Malaysia with the aim of characterizing the PCV2.

To determine the genetic differences among the PCV2 isolates found in Malaysia, the complete genome of PCV2 isolates from thirteen pigs exhibiting PCVAD symptoms in six states was amplified and sequenced. This included three pigs from Selangor (isolate Mal 003/11, Mal004/11, Mal 011/11), three pigs from Melaka (isolate Mal 001/11, Mal 002/11, Mal 008/11), three pigs from Penang (isolate Mal 005/11, Mal 007/11, Mal 013/11), two pigs from Sarawak (isolate Mal 006/11, Mal 009/11), one pig from Perak (isolate Mal 010/11) and finally one pig from Johor (isolate Mal 012/11). All the PCV2 isolates sequenced were closely related to each other displaying between 97-99% nucleotide sequence identity. When compared with other sequences from Genbank (Table 1), all thirteen isolates had closest relationship to isolate AY484407 from Netherlands with nucleotide sequence identity score ranging from 98.3-99.2% (Table 2). The sequences in Table 1 were selected to represent the various subtypes and also to investigate the possible connection between countries of origin from which Malaysia imports the breeder pigs.

**Table 1:** PCV2 isolates used in this study and other isolates reported previously

Genotype	Name	Geographic Location	Reference or source
PCV2b	Mal 001-JF690923	Malaysia	This study
	Mal 002-JF690911	Malaysia	This study
	Mal 003-JF690912	Malaysia	This study
	Mal 004-JF690913	Malaysia	This study
	Mal 005-JF690914	Malaysia	This study
	Mal 006-JF690915	Malaysia	This study
	Mal 007-JF690916	Malaysia	This study
	Mal 008-JF690917	Malaysia	This study
	Mal 009-JF690918	Malaysia	This study
	Mal 010-JF690919	Malaysia	This study
	Mal 011-JF690920	Malaysia	This study
	Mal 012-JF690921	Malaysia	This study
	Mal 013-JF690922	Malaysia	This study

PCV1	U49186	Ireland	Genbank

PCV1/2a	FJ655419	Canada	Genbank
	FJ790425	Canada	Genbank
	FJ655418	Canada	Genbank

PCV2a	DQ629113	USA	Genbank
	DQ629114	USA	Genbank
	AF454546	Korea	Genbank
	AF408635	Canada	Genbank
	AB072301	Japan	Genbank
	AF520783	Korea	Genbank
	AF264038	USA	Genbank
	AY181948	China	Genbank
	AF465211	Taiwan	Genbank

PCV2b	AY484407	Netherlands	Genbank
	HQ831530	Portugal	Genbank
	FJ905469	Korea	Genbank
	HQ831532	Portugal	Genbank
	EU418627	China	Genbank
	GU247989	China	Genbank
	FJ905470	Korea	Genbank
	AY682992	China	Genbank
	AY691169	China	Genbank

PCV2c	EU148503	Denmark	Genbank
	EU148504	Denmark	Genbank
	EU148505	Denmark	Genbank

PCV2d	EF524539	China	Genbank
	EF675241	China	Genbank
	AY181946	China	Genbank
	AY510375	China	Genbank
	AY181947	China	Genbank
	FJ158607	China	Genbank
	AY713470	Germany	Genbank
	DQ151643	China	Genbank
	DQ322701	China	Genbank

**Table 2:** Sequence identity matrix of the capsid protein (ORF2) and the nucleotide alignment of the whole genome of the PCV2 isolates found in Malaysia

Sequences	1	2	3	4	5	6	7	8	9	10	11	12	13	14	15	16	17	18	19	20	21	22
(1) AY484407 PCV2b		98.4	98.6	98.4	98.6	99.2	99.3	98.4	99.1	99	98.3	98.4	98.6	98.3	99.5	99.5	99.2	99.2	99.6	99.7	99.5	76.2

(2) JF690912 Mal003/11	98.7		99.6	99.7	99.5	97.7	98	97.5	98.9	98.9	99.5	99.7	99.6	99.4	98	98.1	98	97.9	98.3	98.2	98.1	76.5

(3) JF690913 Mal004/11	98.7	98.7		99.6	99.9	97.9	98.3	97.9	99.3	99.3	99.6	99.7	99.7	99.4	98.4	98.5	98.3	98.3	98.6	98.4	98.5	76.4

(4) JF690914 Mal005/11	96.5	96.1	96.5		99.7	97.7	98	97.6	99.1	99	99.6	99.7	99.6	99.5	98.1	98.3	98	98	98.4	98.2	98.3	76.3

(5) JF690915 Mal006/11	97.4	96.9	96.9	94.8		97.9	98.3	97.9	99.4	99.2	99.5	99.6	99.7	99.4	98.4	98.5	98.3	98.3	98.6	98.4	98.5	76.3

(6) JF690916 Mal007/11	96.5	96.1	96.1	94	99.1		98.8	97.9	98.4	98.4	97.6	97.7	98	97.6	98.7	98.7	98.4	98.4	98.8	98.9	98.7	75.5

(7) JF690917 Mal008/11	96.1	95.7	95.7	93.5	98.7	99.5		98.1	98.8	98.7	98	98	98.3	97.9	98.9	98.9	98.5	98.6	99	99	98.8	76

(8) JF690918 Mal009/11	96.5	96.1	96.1	94	98.7	99.5	99.1		98.4	98.3	97.5	97.6	97.8	97.5	98.3	98.4	97.7	98.1	98.5	98.3	98.2	75.4

(9) JF690919 Mal010/11	95.7	95.2	95.2	93.1	97.8	98.7	99.1	99.1		99.8	98.9	99	99.2	98.9	98.9	99	98.5	98.8	99.1	98.9	99	76.4

(10) JF690920 Mal011/11	97.4	96.9	96.9	94.8	99.5	98.7	98.2	99.1	98.2		99.1	99.1	99.3	99	98.8	98.9	98.5	98.7	99	98.9	98.9	76.5

(11) JF690921 Mal012/11	96.1	95.7	95.7	93.5	98.2	99.1	99.5	99.5	99.5	98.7		99.5	99.5	99.6	98	98.1	98	97.9	98.3	98.1	98.1	76.4

(12) JF690922 Mal013/11	96.1	95.7	95.7	93.5	98.2	99.1	99.5	99.5	99.5	98.7	100		99.7	99.6	98.1	98.3	98	98	98.4	98.2	98.3	76.4

(13) JF690923 Mal001/11	96.5	96.1	96.1	94	98.7	99.5	99.1	100	99.1	99.1	99.5	99.5		99.6	98.3	98.4	98.3	98.2	98.5	98.5	98.4	76.4

(14) JF690911 Mal002/11	96.1	95.7	95.7	93.5	98.2	99.1	99.5	99.5	99.5	98.7	100	100	99.5		98	98.1	97.9	97.9	98.3	98.1	98.1	76.3

(15) AY682992 PCV2b	100	98.7	98.7	96.5	97.4	96.5	96.1	96.5	95.7	97.4	96.1	96.1	96.5	96.1		99.5	98.8	99.2	99.6	99.3	99.3	76

(16) EU418627 PCV2b	100	98.7	98.7	96.5	97.4	96.5	96.1	96.5	95.7	97.4	96.1	96.1	96.5	96.1	100		98.8	99.3	99.7	99.3	99.3	76.1

(17) FJ905469 PCV2b	98.7	97.4	97.4	95.2	96.9	97	96.5	97	96.1	96.9	96.5	96.5	97	96.5	98.7	98.7		98.5	98.9	99	99	76.1

(18) FJ905470 PCV2b	99.1	97.8	97.8	95.7	96.9	96.1	95.7	96.1	95.2	96.9	95.7	95.7	96.1	95.7	99.1	99.1	97.8		99.4	99.2	99	75.9

(19) GU247989 PCV2b	99.5	98.2	98.2	96.1	96.9	96.1	95.7	96.1	95.2	96.9	95.7	95.7	96.1	95.7	99.5	99.5	98.2	98.7		99.4	99.4	76.1

(20) HQ831530 PCV2b	100	98.7	98.7	96.5	97.4	96.5	96.1	96.5	95.7	97.4	96.1	96.1	96.4	96.1	100	100	98.7	99.1	99.5		99.3	76.2

(21) HQ831532 PCV2b	99.5	98.5	98.2	96.1	97.8	97	96.5	97	96.1	97.8	96.5	96.5	97.1	96.5	99.5	99.5	99.1	98.7	99.1	99.5		76.4

(22) U49186 PCV1	67.6	66.9	67.7	66.9	67.3	66.8	66.8	65.1	68	68.5	67.3	67.3	67.7	67.3	67.6	67.6	67.2	67.2	67.2	67.6	67.6	

PCV2 isolates found in Malaysia were compared with other PCV2b isolates and PCV1. Sequence identity scores for the other PCVs are not shown. The upper right portion is the sequence identity scores of the nucleotide alignment of the whole genome of PCV's; the lower portion is the amino acid sequence identity scores for the capsid protein (ORF2) of PCV2b. The highest nucleotide similarity scores for the Malaysian isolates are shown in bold.

**Figure 2:**
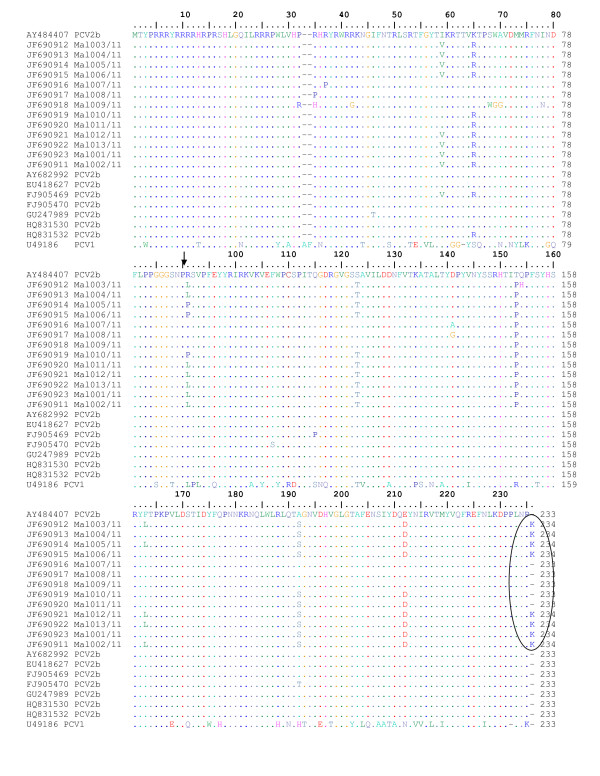
**Amino acid sequence alignment of the putative capsid protein (ORF2) of the PCV2b cluster from this study**. Deletions are indicated by hyphens. Identical sequences are doted. The arrow points at position 88-89. The circled portion denotes the mutations at the stop codon of the putative capsid protein resulting in a lysine (K) residue elongation at the C terminus of the ORF2 [26,28].

Numerous research has been carried out on PCV2 since it is considered to be pathogenic of the two genotypes identified. Amino acid sequence analysis showed that isolates from this study had three distinct clusters. The three clusters were cluster 1C and cluster 1A/1B. Interestingly, 3 of the isolates (isolate Mal 005, Mal006, Mal 010) had a proline (P) encoded at nt. position 89, substituting arginine/leucine (R/L). This mutation pattern has not been reported in other papers. Previous studies have documented that the ORF2 of PCV which codes for the putative capsid protein [28] encodes a protein of 233 amino acid residues. An amino acid sequence comparison revealed that the ORF2 genes of all 13 isolates shared 94.0-100% amino acid sequence identity. One PCV1 (U49816) and 3 PCV1/2a (FJ655419, FJ790425, FJ655418) nucleotide sequence from Genbank was included in this study as out groups. Previous studies have reported that there exist about 63-68% amino acid sequence identity between the ORF2 genes of PCV1 and PCV2 and similarly it was observed that all 13 PCV2 isolates from this study had 65-68% amino acid similarities with the PCV1 (U49816) sequence downloaded from Genbank. All isolates showed about 90-95% sequence similarities with the PCV1/2a sequences (Data not shown). Eight of the isolates (Mal 001/11, Mal 002/11, Mal 003/11, Mal 004/11, Mal 005/11, Mal 006/11, Mal 012/11, Mal013/11) displayed an elongation by one lysine (K) residue at the C-terminus of the putative capsid protein in a mutation at the stop codon of the ORF2 (Figure 2).

**Figure 3:**
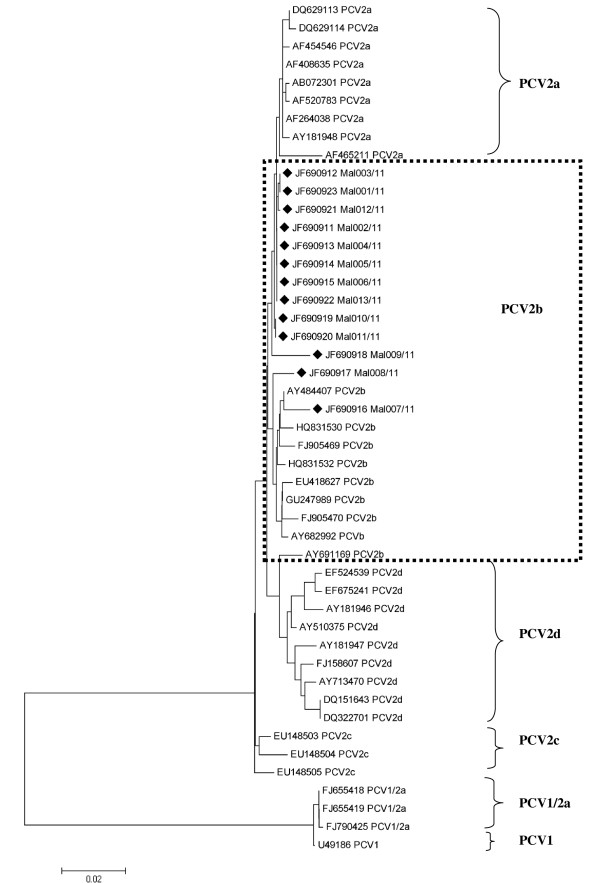
**Phylogenetic analysis of the whole genome of the PCV2 isolates from this study with other sequences from Genbank**. The sequences used to build this tree are cited in Table 1. The phylogenetic tree was constructed using the Neighbour-Joining Method and the image was reduced to 50%. Isolates derived from this study are indicated by the rotated black square. The evolutionary distances were computed using the Maximum Composite Likelihood method. Analyses were conducted using Mega 5.

Phylogenetic analysis confirmed that all thirteen PCV2 isolates in this study grouped together and formed one distinct group with other PCV2b isolates. Likewise, all the PCV2a isolates which are closely related formed another group, while all the PCV1/2a, PCV2c and PCV2d isolates formed their own groups (Figure 3).

Neighboring countries such as Thailand [16] and Indonesia [19] have reported the presence of PCV2b cluster 1C and PCV2b cluster 1A/B respectively suggesting that similar PCV2b clusters as reported in this study are circulating in these few Asian countries in this region. This may also suggest that the swine in this region could be harboring the virus from the same source.

This study showed that 88% of the farms (37 out of 42 farms) were positive for PCV2b which confirms that PCV2 is present in Malaysia. Currently, there are no regulations for the screening of PCV2 or other porcine diseases for imported pigs into the country. Based on statistics provided by the Department of Veterinary Services (DVS), Malaysia, there are some evidence that breeders and swine products are imported from Australia, Canada, Netherlands, America, Vietnam and other countries. Although the present study and statistics available suggests that there may be a link between imports of pig products, however, no definitive conclusions can be made as data on the countries of import from the farms in this study are not available for analysis.

The samples from this study were collected in light of the PCV2 occurrences in Malaysia in which a high number of clinical symptoms were observed in many farms. Taking into account the possibility of virus spread through animal migration and commercial trading, it is expected that viruses in the commercial swine breeds would have multiple genetic signatures. Therefore, understanding the genetic characteristics and diversity of viruses provides new information to guide and prioritize decisions for the control of disease in livestock including the implementation of effective vaccination strategies and good biosecurity programs for the swine industry. All animals are susceptible to a wide range of diseases that may affect its productivity. Among solutions required to minimize the risk of disease spread, strengthening the biosecurity and husbandry practices in a farm is of utmost importance. PCVs are highly resistant to inactivation by common detergents and disinfectants. This makes decontamination of infected premises difficult, if not impossible. But combining accurate diagnosis, removal of diseased animals from farms, routine vaccination programs and a good husbandry practice could be an excellent measure for controlling losses attributed to PCV2 infections.

Each production system requires suitable biosecurity measures and the key to an enhanced biosecurity lies in the perception of biosecurity by the stakeholders in the pig farming industry for a meaningful change to take place.

In the past, a centralized pig farming has been suggested by the authorities to control pollution, safeguard these food animals that are meant for human consumption, improve sanitary condition, minimize environmental problems and organize the production of pigs but unfortunately, the centralized pig farming did not materialize. Perhaps, it is time that the local authorities review these measures to improve livestock production systems and to reduce the risk of disease agents.

## Conclusion

Publication of whole genome sequences allows researchers to trace the presence and the spread of economically important viruses, determine their genetic distribution and assist in making priority decisions for good control programs. The findings of this study suggests that routine screening would be helpful to monitor the status of PCV2 and subsequently its control by implementing a good vaccination program and other measures such as education to the pig farmers on the consequences of PCVAD becoming an endemic disease in the country. More studies need to be done to establish the function of the mutations present in the group 1 (PCV2b) found in Malaysia before any conclusions can be made with regards to its relationship to its pathogenicity.

In conclusion, the 2.2 billion ringgit swine industry in Malaysia is an economic asset to the country's resources. Therefore, all appropriate measures should be taken to manage it well.

## Methods

### Sampling and Screening of PCV1 and PCV2 by PCR

**Figure 4:**
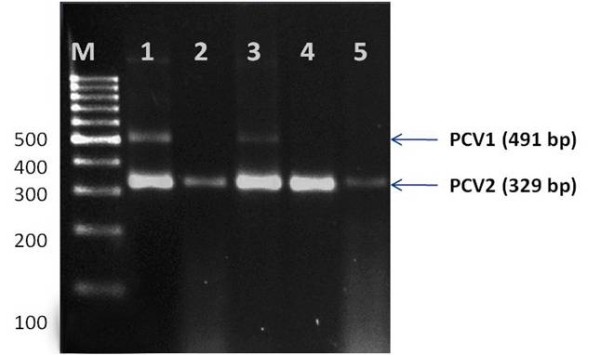
**Gel electrophoresis image of the multiplex PCR used for the screening of PCV1 and PCV2**. The amplicon size for PCV1 is 491 bp and the amplicon size for PCV2 is 329 bp [39].

Organ samples consisting of lymph nodes, kidney, liver, lungs and other specimens such as fecal samples were collected from 42 pig farms in Malaysia from animals displaying classic PCVAD clinical signs. The states included in this study were Penang, Perak, Selangor, Melaka, Johor and Sarawak. The pooled organ samples were subjected to nucleic acid extraction by using Trizol LS reagent according to the standard manufacturer's protocol. Nested multiplex PCR with modifications from a method previously described [39] was established. The primer sets for the first step and nested step are listed in Table 3. Both the PCR mixtures consisted of 2.5 μl of 10X PCR buffer, 1.5 μl 25 mM MgCl_2_, 0.5 μl 10 mM dNTPs, and 0.5 μl of 5 U *Taq *Polymerase, DNA template, the respective primer sets and PCR grade water to make up the final volume of 50 μl per reaction. The PCR tubes were subjected to PCR amplification in a thermocycler (MyCycler™, Bio-Rad). The DNA fragment sizes were determined via agarose gel electrophoresis. This multiplex PCR is able to distinguish PCV1 and PCV2 (Figure 4).

**Table 3:** Primer pairs used for screening the suspected cases and for sequencing the complete genome of PCV2

Primer	Sequence (5'-3')	Size of PCR Product (bp)	Nucleotide position	PCR step	Reference
PCV-1 forward	CTC GGC AGC GTC AGT GAA AA	570	800-819	First step (xPCR)	[39]
				
PCV-1 reverse	AAA TTA CGG GCC CAC TGG CT		1350-1369		
		
PCV-2 forward	CGG ATA TTG TAG TCC TGG TCG	481	1095-1115		
				
PCV-2 reverse	ACT GTC AAG GCT ACC ACA GTC A		1549-1570		
	
PCV-1 forward	CCT TCC GAG GAG GAG AAA AAC	491	879-900	Nested PCR (nPCR)	
				
PCV-1 reverse	AAA TTA CGG GCC CAC TGG CT		1350-1369		
		
PCV-2 forward	GGT TTG GGT GTG AAG TAA CGG G	329	1242-1263		
				
PCV-2 reverse	ACT GTC AAG GCT ACC ACA GTC A		1549-1570		

CV1-forward	AGG GCT GTG GCC TTT GTT AC	989	1336-1355	Sequencing	[10,23]
				
CV2-reverse	TCT TCC AAT CAC GCT TCT GC		536-556		
		
CV3-forward	TGG TGA CCG TTG CAG AGC AG	1093	453-471		
				
CV4-reverse	TGG GCG GTG GAC ATG ATG AG		1525-1544		

All pigs were humanely slaughtered for sample collection under the supervision of veterinarians from the Faculty of Veterinary Medicine, UPM. There was no experimental research done on the pigs.

### PCR amplification of the complete genome of PCV2

**Figure 5:**
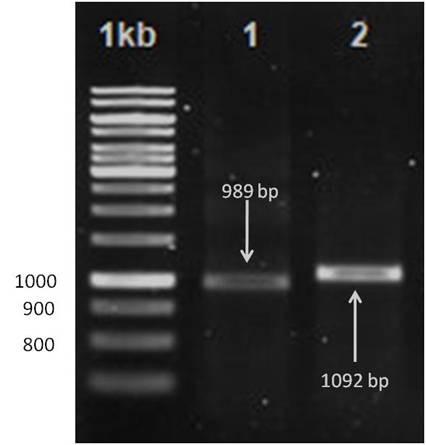
**Gel electrophoresis images of the 2 overlapping fragments that amplify the whole PCV2 genome **[10,23]. The amplicon sizes for the two overlapping fragments are 989 bp (Lane 1) and 1092 bp (Lane 2).

A published primer was used for amplifying the whole genome of PCV2. The primer sets were able to amplify two overlapping fragments that represent the entire genome of PCV2. The first set of primers, CV1 and CV2, amplifies a 989-bp fragment, and the second set of primers, CV3 and CV4, amplifies a 1,092 bp fragment (Table 3). The PCR amplification of the complete genome of PCV2 was established with modifications from the method as described by Fenaux M et al. 2000 (Figure 5) [10,23].

### Nucleotide sequencing, sequencing analysis and construction of phylogenetic tree

The PCR products of the expected sizes were purified using the PCR clean-up gel extraction kit according to the manufacturer's protocol with slight modifications (Macherey-Nagel, Germany). Sequencing of the complete genome of PCV2 was done in a commercial sequencing facility using the BigDye Terminator v3.1 cycle sequencing kit. After sequencing, a Basic Local Alignment Search Tool (BLAST) was performed with the derived DNA sequence as a preliminary measure to confirm that all samples were true PCV2 when compared with other sequences deposited in Genbank. The sequence editing and assembly were done by using CLC Workbench. Multiple sequence alignments were done by using ClustalW. The phylogenetic tree was constructed by using the distance-based neighbor joining method and generated by using Mega 5 (Biodesign Institute, Tempe, Arizona) and evaluated using the bootstrapping method calculated on 1000 repeats of the alignment (Data not shown). The sequence identity matrix data was generated with BioEdit Sequence Alignment Editor version 7.0.5.2 (Tom Hall, US). Sequences used for constructing the phylogenetic tree are listed in Table 1. PCV1 sequences were included in the tree as an out group, whilst PCV1/2a sequences were included to investigate whether similar recombinant events of the ORF1 and ORF2 of PCV2 are found in Malaysia. Sequences for PCV2e could not be analyzed as the sequence was not available in Genbank.

### Nucleotide sequence accession numbers

The complete genomic sequences of the 13 PCV2 isolates reported in this paper were deposited with the GenBank database under accession numbers JF690911, JF690912, JF690913, JF690914, JF690915, JF690916, JF690917, JF690918, JF690919, JF690920, JF690921, JF690922 and JF690923. These sequences are downloadable from Entrez Pop Set data as a group of sequences.

## Competing interests

The authors declare that they have no competing interests.

## Authors' contributions

SJ designed and conceived the research, participated in the conceptual aspect of the work, performed the experiments and wrote the manuscript. OPT, PLY, TDY, CPY and LBK provided consultation and coordination. All authors read and approved the final manuscript.
